# In Memory of Professor Neil J. Smart PhD FRCS

**DOI:** 10.3389/jaws.2025.15161

**Published:** 2025-07-10

**Authors:** Dominic Slade

**Affiliations:** Salford Royal NHS Foundation Trust, Salford, United Kingdom

**Keywords:** research, hernia, parastomal, UEMS, examiner

It is with profound sadness that we announce the passing of our friend and colleague, Professor Neil Smart, whose extraordinary life and career left an indelible mark on all who had the privilege of knowing him. As a trainee in Exeter, Devon in 2009, he helped develop a pivotal decade and a half of surgical research and practice during his time as surgical registrar, fellow, and consultant surgeon. His passion in wanting to learn, travel and absorb best practice, then transfer this through mentorship of trainee surgeons encouraging them to read, write, travel, talk and solve problems collaboratively formed the principles that underpinned all that he did.

Neil was a man of immense intellect, humility, and generosity. In a world where many build their empires by self-promotion and climbing over others, Neil did the opposite, he brought everyone else up with him. Everyone was met with his characteristic kindness, humour, respect, and encouragement. His wise words will echo through hospital corridors and lecture halls, passed down to generations of trainees inspired by his example.

For over a decade, Neil was deeply committed to the European Hernia Society, playing a pivotal role in shaping its direction and advancing its mission. He contributed meaningfully to the development of clinical guidelines, dedicated himself to educating surgeons in the complex field of abdominal wall surgery, and worked tirelessly to elevate the society’s scientific standing. His thoughtful leadership and tireless work ethic left an enduring legacy that will continue to guide and benefit the surgical community for many years to come.

Neil was also a passionate advocate for patient-centred care. He championed the inclusion of patient and nursing representatives on surgical committees and was determined to ensure their voices were heard in clinical decision-making and research. He was especially committed to those living with parastomal hernia, working to understand their quality-of-life concerns and pushing for better recognition, support, and treatment. He did this through the best way he knew-the creation of not one but two important multicentre studies (PROPHER and CIPHER), and an EHS course which he chaired in April whilst also undergoing chemotherapy. His empathy and advocacy helped redefine what compassionate, holistic surgical care should look like.

The outpouring of love and affection from across the UK, Europe, and the USA on his passing is a testament to the lives he touched. His mentorship helped shape countless careers, including many current board members of the European Hernia Society, a fitting reflection of the influence he wielded not through ego, but through empowerment. Even in illness, Neil’s sharp wit never faltered. As a patient himself in Newcastle, he met a visiting upper GI multidisciplinary team and true to form offered them a few suggestions on how they may improve. He joked afterward, “I think I put my operating team through hell, the surgeon has aged a decade.”

Neil’s life was a masterclass in compassion, leadership, and integrity. He lives on in the many people he supported not just through what he taught, but through how he made others feel: seen, valued, and capable of more than they believed possible.

We will all miss him dearly.

## Data Availability

The original contributions presented in the study are included in the article/supplementary material, further inquiries can be directed to the corresponding author.

